# Detection and Quantification of the Epstein-Barr Virus in Lymphoma Patients from Ethiopia: Molecular and Serological Approaches

**DOI:** 10.3390/microorganisms11102606

**Published:** 2023-10-22

**Authors:** Seifegebriel Teshome, Kidist Zealiyas, Abdulaziz Abubeker, Fisihatsion Tadesse, Jayalakshmi Balakrishna, Christoph Weigel, Tamrat Abebe, Elshafa Hassan Ahmed, Robert A. Baiocchi

**Affiliations:** 1 Department of Microbiology, Immunology and Parasitology, Addis Ababa University, Addis Ababa 9086, Ethiopia; seifegebriel.teshome@aau.edu.et (S.T.); tamrat.abebe@aau.edu.et (T.A.); 2 Ethiopian Public Health Institute (EPHI), Addis Ababa 1242, Ethiopia; kzealiyas@gmail.com; 3 Aklilu Lemma Institute of Pathobiology, Addis Ababa University, Addis Ababa 1176, Ethiopia; 4 Department of Internal Medicine, Addis Ababa University, Addis Ababa 9086, Ethiopia; abdulazizas88@gmail.com (A.A.); fishtadat@yahoo.com (F.T.); 5 Department of Pathology, College of Medicine, The Ohio State University, Columbus, OH 43210, USA; jayalakshmi.balakrishna@osumc.edu; 6 Division of Hematology, Department of Internal Medicine, College of Medicine, The Ohio State University, Columbus, OH 43210, USA; christoph.weigel@osumc.edu; 7 Comprehensive Cancer Center, The James Cancer Hospital and Solove Research Institute, The Ohio State University, Columbus, OH 43210, USA

**Keywords:** Epstein-Barr virus, lymphoma, *EBNA1*, qPCR, VCA IgG, Ethiopia

## Abstract

The Epstein-Barr virus (EBV) is a known oncogenic virus associated with various lymphoma subtypes throughout the world. However, there is a lack of information regarding EBV prevalence in lymphoma patients, specifically in Ethiopia. This study aimed to investigate the presence of the EBV and determine its viral load in lymphoma patients from Ethiopia using molecular and serological approaches. Lymphoma patient samples were collected from the Ethiopian population. DNA and serum samples were extracted and subjected to molecular detection methods, including quantitative polymerase chain reaction (qPCR) analysis targeting the *EBNA1* gene. Serological analyses were performed using an enzyme-linked immunosorbent assay (ELISA) to detect EBV viral capsid antigen IgG antibodies. EBV DNA was detected in 99% of lymphoma patients using qPCR, and serological analyses showed EBV presence in 96% of cases. A high EBV viral load (>10,000 EBV copies/mL) was observed in 56.3% of patients. The presence of high EBV viral loads was observed in 59.3% of HL patients and 54.8% of NHL patients. This study provides important insights into the prevalence and viral load of the EBV among lymphoma patients in Ethiopia. The findings contribute to the limited knowledge in this area and can serve as a foundation for future research.

## 1. Introduction

The EBV is an oncogenic, gamma herpes virus associated with various lymphoid and epithelial malignancies. The virus has infected more than 90% of people worldwide [1]. Primary infection with the EBV can lead to the manifestation of infectious mononucleosis (IM). However, the majority of individuals with a healthy immune system respond effectively, developing robust immunity and remaining asymptomatic. The EBV is known to be associated with the development of various lymphoid and epithelial malignancies. These include Burkitt lymphoma (BL), Hodgkin’s lymphoma (HL), nasal T/NK lymphomas, nasopharyngeal carcinoma (NPC), and certain cases of gastric carcinoma [2]. The virus’s involvement in these malignancies underscores the significance of understanding its role in disease progression and the potential for targeted interventions and treatments [3]. 

The EBV is primarily transmitted through saliva, and the presence of the infectious virus can be detected in the oropharyngeal secretions of individuals with IM, as well as those who are immunosuppressed. In addition, lower levels of the virus can also be found in the oropharyngeal secretions of healthy individuals who have previously been infected with the EBV and have seropositivity for the virus [4]. The mechanisms of oncogenesis of the EBV can vary depending on the specific type of tumor. However, certain fundamental characteristics of EBV biology are observed across various EBV-associated cancers [5]. The initial stage in EBV tumorigenesis involves the establishment of a persistent or latent infection. During this phase, the virus remains in a dormant state within the host cells, allowing it to evade the immune system and persist over an extended period [6]. 

The EBV latent state is a highly active, dynamic, and “programmed” process, where the viral genome expresses a restricted set of latency-associated genes [7]. These latent genes include six Epstein-Barr nuclear antigens (*EBNAs*) *EBNA1*, *EBNA2*, *EBNA3A*, *EBNA3B*, *EBNA3C*, and *EBNA* leader protein (*EBNA-LP*) and three latent membrane proteins (*LMPs*), *LMP1*, *LMP2A*, and *LMP2B* [8]. In addition to the expression of viral genes, the EBV also produces noncoding RNAs, namely EBV-encoded small RNAs (*EBERs*), specifically *EBER-1* and *EBER-2* during latency. These noncoding RNAs play important roles in the viral life cycle and may contribute to the oncogenic potential of the EBV [9]. There are around 44 known viral microRNAs (miRNAs) [10]. 

The detection of the EBV can be performed by using Epstein-Barr virus-encoded RNA in situ hybridization (EBER-ISH), serology (viral IgG and IgM ELISA), and molecular techniques using different polymerase chain reaction methods (PCR). Each technique has its own pros and cons, but EBER-ISH is considered a gold standard for EBV detection in pathology specimens. Technically, the PCR technique is also a rapid and sensitive test crucial for viral quantification [11,12,13,14]. In our study, both serological and molecular methods were employed to assess the presence and quantification of the EBV in lymphoma patients in Ethiopia. By combining these two complementary approaches, our study aimed to comprehensively investigate the presence, serostatus, and viral load of the EBV in lymphoma patients in Ethiopia. 

While the EBV is ubiquitous worldwide, geographic variability in the incidence of EBV-related tumors has been described [15]. However, the scarcity of published data on the molecular analysis of the EBV in sub-Saharan Africa, particularly in the East African region, underscores the significance of our research findings. Thus, our study fills an important scientific gap by providing the first evidence-based data on the prevalence and viral load of the EBV in lymphoma patients in Ethiopia. Therefore, this study aims to assess the prevalence of the EBV in lymphoma patients using molecular techniques, to quantify the viral load of the EBV in lymphoma patients from Ethiopia, to evaluate the serological response to EBV infection in lymphoma patients, and to explore the association between EBV load and lymphoma subtypes in Ethiopian patients.

## 2. Materials and Methods

### 2.1. Study Design and Participants

This cross-sectional study was conducted at Tikur Anbessa Specialized Hospital (TASH) in Addis Ababa, Ethiopia, spanning from July 2020 to March 2022. TASH is the largest and the oldest referral hospital that provides different medical services for patients coming from all over the country. Both retrospective and prospective study designs were employed to collect data. Retrospective data collection involved obtaining formalin-fixed paraffin-embedded (FFPE) lymphoma tissue blocks from the pathology department at TASH. Prospective data collection involved enrolling confirmed lymphoma patients from the hematology clinics at TASH. Additionally, suspected lymphoma patients were included from the minor surgery unit, specifically those scheduled for lymph node surgery to confirm the presence of lymphoma. 

### 2.2. Data and Sample Collection

To collect the FFPE lymphoma blocks, we reviewed a two-year period of results (from 2019 to 2020) from the pathology department. All confirmed lymphoma patients who had FFPE blocks available, as determined by the pathologist, were included in the study. Sociodemographic and clinical data with a pathology report were also obtained using the check list. Two sections of 5 μm thickness were collected from the FFPE blocks. These sections were placed in 1.5 mL Eppendorf tubes and stored at −20 °C to preserve the genomic DNA until the DNA extraction process was performed. 

For the prospective study groups, the first group was confirmed lymphoma patients, and we collected around 20 mL of venous blood using EDTA and SST tubes. The collected blood was used to isolate peripheral blood mononuclear cells (PBMCs), plasma, and serum. For the second group, which consisted of suspected lymphoma patients, a fresh lymph node biopsy tissue sample (approximately 5 mm^3^) was obtained through surgery. This tissue sample was stored in 1% phosphate-buffered saline (PBS) immediately after surgery. Additionally, a 10 mL venous blood sample was collected from the suspected lymphoma patients to obtain a serum sample. Alongside the sample collection, sociodemographic data, including age, sex, lymphoma status, and other relevant clinical information, were also collected using a structured questionnaire.

### 2.3. Sample Processing

Isolation of PBMCs was performed using the Ficoll–Paque technique (Global Life Sciences Solutions, Marlborough, MA, USA). Ficoll–Paque medium is a density gradient medium that allows for the separation of different cellular components based on their density. To isolate lymph node mononuclear cells (LMNCs) from fresh lymph node biopsies, a single cell suspension technique was utilized. This technique involves the dissociation of the tissue into individual cells, allowing for the separation and isolation of specific cell populations. Viability and cell count assessments were performed on both the isolated PBMCs and LMNCs using the trypan blue staining procedure. The majority of the samples showed greater than 90% viability, indicating that the isolated cells were of good quality for further analysis. After isolation, both PBMCs and LMNCs were stored at −80 °C in 10% dimethyl sulfoxide (DMSO) (Thermo Fisher Scientific, Waltham, MA, USA) until DNA extraction was performed. To collect serum, the blood samples were allowed to clot, and then the serum and plasma were isolated from the blood samples by centrifugation at 1500× *g* for 10 min. The isolated serum and plasma samples were then stored at −80 °C in a freezer until further analysis.

### 2.4. Extraction of Genomic DNA

Genomic DNA was extracted from FFPE blocks using a QIAamp DSP DNA FFPE tissue kit (QIAGEN, Hilden, Germany) after applying the deparaffinization solution (ready to use) (QIAGEN, Hilden, Germany) on the FFPE blocks according to the manufacturer’s instructions. DNA from PBMCs and LMNCs was extracted using a QIAamp mini-DNA kit (QIAGEN, Hilden, Germany) according to the manufacturer’s instructions. The extracted DNA’s concentration and quality were measured using a Qubit fluorometer (Thermo Scientific, Waltham, MA, USA) and a Nanodrop spectrophotometer (Thermo Fisher Scientific, Waltham, MA, USA), respectively. The extracted DNA from all three sample types was stored at −80 °C until further analysis was performed.

### 2.5. EBV Serology Detection Using VCA IgG Antibody through ELISA

To determine the presence of EBV IgG antibodies, we used BioPlex 2200 EBV IgG kits on a BioPlex 2200 Analyzer (Bio-Rad, Hercules, CA, USA) according to the manufacturer’s instructions. Serum samples collected from the study participants were used for EBV serological detection and the results of the EBV IgG antibodies were classified based on the antibody index (AI). The interpretation of the results was as follows: AI ≤ 0.8 AI was negative, AI 0.9–1 AI was equivocal (intermediate), and AI ≥ 1.1 AI was positive. To validate the accuracy of the test, internal positive and negative controls were also performed.

### 2.6. EBV Detection and Quantification Using Real-Time qPCR

Detection and quantification of the EBV was determined by amplifying the target region of the *EBNA1* gene. The amplification of the *EBNA1* gene region was performed using a ViiA 7 real-time qPCR system (Applied Biosystems, Waltham, MA, USA). We used 10 ng of DNA sample as the initial concentration for the qPCR reaction and the qPCR was carried out using a Fast SYBR green master mix (Applied Biosystems, Waltham, MA, USA). Specific primer sequences designed to target the *EBNA1* gene locus (forward: TCATCATCATCCGGGTCTCC, reverse: CCTACAGGGTGGAAAAATGGC) were used, and signals were normalized to host genome DNA using primers specific for human actin beta (*ACTB)* locus (forward: CAGGCAGCTCGTAGCTCTTC, reverse: TCGTGCGTGACATTAAGGAG). The reaction was performed using 5 μL of 2×master mix, 0.25 μL of forward (10 μM) and 0.25 μL of reverse (10 μM) primers, 2.5 μL of PCR-grade water, and 2 μL of DNA at a concentration of 5 ng/μL.

### 2.7. qPCR Thermal Reaction and Standard Curve Preparation

The real-time qPCR protocol consisted of 40 cycles of amplification for denaturation at 95 °C for 1 s, annealing at 60 °C for 20 s, and extension at 70 °C for 30 s. DNA extracted from the Raji cell line was used as a positive control and K-562 cell line DNA was used as a negative control. To quantify the EBV viral load, twelve standards of known DNA copy numbers from the *EBNA1* gene and human *ACTB* gene products were used. The *EBNA1* gene standard was obtained from *EBNA1* amplicon with accession number EBNA1-NC_007605.1 and actin beta obtained from Homo Sapiens with the accession number NM_001101.5 was used for the *ACTB* standard. These standards were prepared by serial dilution, creating a range of concentrations. The purpose of these standards was to establish a standard curve, which relates the cycle threshold (CT) values of the qPCR amplification to the known DNA copy numbers. The CT values obtained from the standards were used to construct the standard curve and this standard curve serves as a reference to determine the DNA copy number in samples based on their CT values. Each sample and standards were run in triplicate with 384-well PCR plates to ensure the reproducibility and to reduce the potential variability in the results. If there was a significant disparity between the CT values within the triplicates, we retested the sample to confirm the results.

### 2.8. Data Analysis 

The data were analyzed using SPSS version 26.0 for data entry and analysis of the pathological, serological, and molecular data. Descriptive statistics were calculated to summarize the data, and the association between the detection and viral load of the EBV with other sociodemographic and clinical variables was assessed using the Pearson Chi-square test. Associations with p-values less than 0.05 were considered statistically significant.

### 2.9. Ethical Clearance

Ethical approval was obtained from the departmental ethical approval committee, the College of Health Sciences Institutional Review Board at Addis Ababa University (protocol number: 056/21/DMIP) on 8 November 2021, and the National Research Ethical Review Committee on 23 September 2022.

## 3. Results

### 3.1. Characterstics of Study Participants

A total of 305 participants were enrolled in this study. Among them, 133 individuals were included based on retrospectively collected FFPE lymphoma blocks, while 120 participants were confirmed lymphoma patients. The remaining 52 participants were suspected to have lymphoma.

In terms of gender distribution, *n* = 197 (64.6%) of the study participants were male, resulting in a male-to-female ratio of 1.8 to 1. The age of the participants varied from 3 to 85 years, with a mean age of 38 ± 17.5 years (standard deviation). The age group between 41 and 50 years had the highest number of participants *n* = 69 (22.6%). Sociodemographic and clinical profiles of the study participants are provided in Table 1. 

### 3.2. Histopathological Profiles

Out of the total 305 study participants, 288 individuals were diagnosed with lymphoma based on confirmed pathology reports. On the other hand, 17 patients initially suspected to have lymphoma were found to be negative for the disease after obtaining a pathology report. Consequently, these non-lymphoma patients were excluded from further analyses.

**Figure 1:**
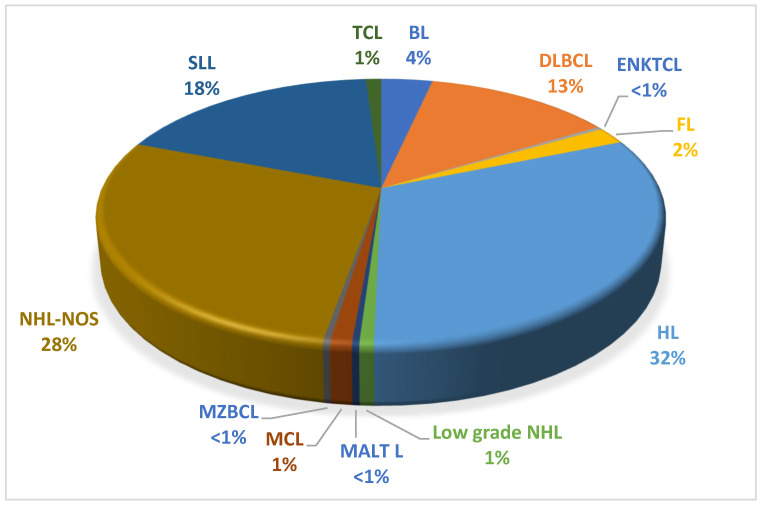
Histologic subtypes of lymphoma in all study participants (*n* = 288). Abbreviations: DLBCL: diffuse large B cell lymphoma; BL: Burkitt lymphoma; HL: Hodgkin’s lymphoma; NHL: non-Hodgkin’s lymphoma—not otherwise specified; SLL: small lymphocytic lymphoma; FL: follicular lymphoma, MCL: mantle cell lymphoma; MALT L: mucosa-associated lymphoid tissue lymphoma; MZBCL: marginal zone B cell lymphoma; ENKTCL: extranodal natural killer/T cell lymphoma; TCL: T cell lymphoma.

Among the 288 lymphoma patients, *n =* 91 (32%) were classified as Hodgkin’s lymphoma patients and *n =* 197 (68%) were categorized as non-Hodgkin’s lymphoma patients. Within the NHL subgroup, the majority of cases were small lymphocytic lymphomas (SLLs), accounting for 18% (*n* = 52) of the total NHL patients. Other NHL subtypes included diffuse large B cell lymphoma (DLBCL) with 13% (*n* = 37), Burkitt lymphoma (BL) with 4% (*n* = 10), follicular lymphoma (FL) with 2% (*n* = 6), mantle cell lymphoma (MCL) with 1% (*n* = 3), T cell lymphoma (TCL) with 1% (*n* = 3), low grade lymphoma with 0.7% (*n* = 2), mucosa-associated lymphoid tissue lymphoma (MALT L) with 0.3% (*n* = 1), marginal zone B cell lymphoma (MZBCL) with 0.3% (*n* = 1), extranodal natural killer/T cell lymphoma (ENKTCL) with 0.3% (*n* = 1), and the remaining cases were NHL subtypes not otherwise classified(*n* = 81.28%) (Figure 1).

**Figure 2:**
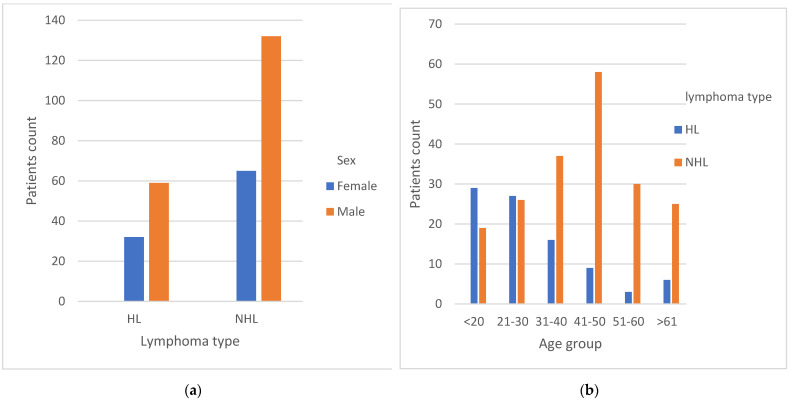
Distribution of sex and age groups with the lymphoma types. (**a**) The distribution of different lymphoma types with sex; (**b**) the distribution of different lymphoma types with age group. Abbreviations: HL: Hodgkin’s lymphoma; NHL: non-Hodgkin’s lymphoma.

There was a higher prevalence of male patients in both HL and NHL groups. Among the 91 HL patients, 64.8% (*n* = 59) were male, while among the 197 NHL patients, 67% (*n* = 132) were male (Figure 2a). HL was more predominant in the age group younger than 20 years old, accounting for 60.4% (*n* = 29) of cases within that age range. On the other hand, a higher proportion of NHL patients were found in the age group of 51–60 years old, comprising 91% (*n* = 30) of cases within this age group (Figure 2b). Out of the total HL patients, 9.9% (*n* = 9) were found to be HIV-positive. Similarly, among the NHL patients, 8.6% (*n* = 20) were HIV-positive. Among the NHL patients who were HIV-positive, 45% (*n* = 9) of them were diagnosed with DLBCL.

### 3.3. Serological Detection of EBV

A total of 164 serum samples were collected from both suspected and confirmed lymphoma patients to test for EBV VCA IgG. Among these samples, 150 were subjected to serological analysis (49 from HL patients and 101 from NHL patients). The remaining 14 samples were excluded from further analysis due to non-lymphoma reports obtained from pathologies of suspected patients cohort groups.

**Figure 3:**
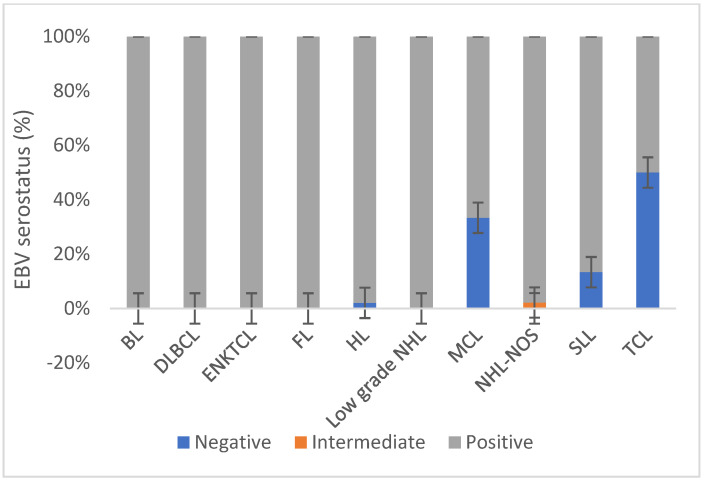
Serological detection of the EBV among different lymphoma types in Ethiopia. Abbreviations: DLBCL: diffuse large B cell lymphoma; BL: Burkitt lymphoma; HL: Hodgkin’s lymphoma; NHL-NOS: non-Hodgkin’s lymphoma—not otherwise classified; SLL: small lymphocytic lymphoma; FL: follicular lymphoma, MCL: mantle cell lymphoma, ENKTCL: extranodal natural killer/T cell lymphoma; TCL: T cell lymphoma; Low grade NHL: Low grade non-Hodgkin’s lymphoma.

Out of the 150 serum samples analyzed, 96% (*n* = 144) of the study participants were tested as positive for the EBV IgG antibody. However, 3.3% (*n* = 5) tested negative and 0.07% (*n* = 1) showed an intermediate result for EBV IgG antibodies, respectively. From the different lymphoma types, EBV IgG was detected in the serum of 98% (*n* = 48) of HL patients and 95% (*n* = 96) of NHL patients. Among the different types of NHL, it was observed that some patients with T cell lymphoma (TCL), mantle cell lymphoma (MCL), and small lymphocytic lymphoma (SLL) were EBV seronegative. Specifically, out of the total number of TCL patients, one out of two individuals were found to be EBV seronegative. Similarly, among MCL patients, one out of three patients lacked EBV seropositivity. Among SLL patients, 2 out of 13 individuals were EBV seronegative. There was no statistical significance between the HL and NHL types and EBV serology was observed (*p*-value = 0.64) (Figure 3).

Regarding gender distribution, 95.8% of male participants and 96.3% of female participants were seropositive for the EBV and there was no statistically significant association between sex and EBV serology (*p*-value = 0.31). Among the 28 HIV-positive study participants, 26 had their EBV serology tested, and all of them (100%) were found to be EBV seropositive. Two serum samples were not collected from HIV-positive patients from the suspected groups due to unwillingness to give their blood sample. Among the 106 HIV-negative patients, 94.3% (*n* = 100) were seropositive for the EBV. All 18 patients who were unaware of their HIV status were seropositive for the EBV and there was no significant association between HIV status and EBV serological positivity (*p*-value = 0.63). Table 2 summarizes the serological findings related to EBV infection within various datasets.

### 3.4. EBV DNA Quantification Using Real-Time qPCR

Among the 288 lymphoma patients, the EBV was detected in 99% (*n* = 285) of the cases. The quantification of EBV copies/mL ranged from 10^2^ to 10^9^ copies/mL, with a median viral load count of approximately 1.1 × 10^3^ EBV copies/mL. Based on the limit of detection of the assay using the negative control, the EBV viral copy numbers were categorized into three groups: high, low, and very low. A high EBV viral load (>10,000 EBV copies/mL) was observed in 56.3% (*n* = 162) of the study participants, while 16% (*n* = 46) had a low EBV viral load (5000–10,000 EBV copies/mL) and 27.8% (*n* = 80) had a very low EBV viral load (<5000 EBV copies/mL).

When analyzing different lymphoma types, a high EBV viral load was found in 59.3% (*n* = 54) of Hodgkin’s lymphoma patients and 54.8% (*n* = 108) of non-Hodgkin’s lymphoma patients. Among NHL patients, those with DLBCL had the highest prevalence of high EBV viral loads, with 67.6% (*n*= 25) of the 37 DLBCL patients exhibiting high EBV viral loads. This was followed by BL, with 50% (*n* = 5) of 10 cases, SLL with 46% (*n* = 24) of 52 cases, and FL with 33.3% (*n* = 2) of 6 cases. However, no significant association was observed between different lymphoma types and EBV viral loads (*p*-value = 0.11).

In terms of sample types, tissue biopsy samples collected from suspected lymphoma patients showed a higher EBV viral load compared to retrospectively collected FFPE blocks and blood samples obtained from confirmed lymphoma patients. Among the samples, 94.3% of DNA extracted from LMNCs had a high EBV viral load. However, 72.5% of the DNA extracted from PBMC samples and 31.6% of DNA from FFPE lymphoma block samples exhibited a high EBV viral load (>10,000 EBV copies/mL). There was a significant association between different sample types and EBV viral loads (*p*-value < 0.001). Specifically, DNA isolated from lymph node biopsies had a higher EBV viral copy number compared to DNA samples extracted from blood and FFPE block samples.

The analysis also revealed that high EBV viral copy numbers were detected in 56% (*n* = 107) of male participants and 56.7% (*n* = 55) of female participants. However, no significant association between sex and EBV viral load was found (*p*-value = 0.99). Furthermore, among the different age groups, 73.5% (*n* = 39) of individuals in the young age group (21–30 years) exhibited a higher EBV copies/mL value compared to the other age groups. Nevertheless, there was no significant association between age groups and EBV viral loads (*p*-value = 0.49). Among the 28 lymphoma patients who were HIV-positive, 85.7% (*n* = 24) had a high EBV viral load. Similarly, among the 109 lymphoma patients who were HIV-negative, approximately 73.4% (*n* = 80) had a high EBV viral load. There was a significant association between HIV positivity in lymphoma patients and a higher EBV viral load (*p*-value < 0.01) (Table 3).

## 4. Discussion

EBV infection is widespread among the global population and is linked to various EBV-associated malignancies. The majority of these malignancies arise from the infection of B or T lymphocytes as well as epithelial cells. The detection of the EBV through serological and molecular techniques plays a critical role in disease monitoring, prognosis assessment, and the implementation of preventive measures [11]. Hence, investigating the burden of the EBV among lymphoma patients and other population groups from diverse regions worldwide is essential for comprehending the impact and prevalence of these diseases within different communities. Developing countries such as Ethiopia often lack evidence-based studies in this regard, making this study particularly significant as it is the first to determine the molecular and serological prevalence of the EBV among lymphoma patients in the country.

Previous studies have established that over 90% of the global population is infected with the EBV [16,17]. Our study similarly found the serological prevalence of the EBV among Ethiopian lymphoma patients to be 95.2%. This finding aligns with other studies and reports conducted worldwide [18]. Moreover, our results indicated a higher prevalence of EBV VCA IgG antibodies in male individuals, HIV-positive patients, and those diagnosed with HL.

The pathological findings in our study indicated a higher prevalence of both Hodgkin’s lymphoma (HL) and non-Hodgkin’s lymphoma (NHL) among male patients, which is consistent with findings from other studies conducted globally [19,20,21]. Additionally, our study revealed that HL was more predominant in the age group of less than 20 years. This finding aligns with observations made in studies conducted in Zambia [22] and Iraq [23]. However, in developed countries, studies have shown that the incidence of HL is highest in individuals above the age of 60 [24]. This disparity is primarily attributed to factors such as poor socioeconomic status in developing countries, hygienic conditions, and genetic variations [21,25].

Our study also demonstrated the molecular presence and quantification of the EBV in different lymphoma types. High-viral-load (>10,000 EBV copies/mL) EBV was detected in 56.3% of the study participants, which is comparable to similar studies conducted in Nigeria (54.5%) [26], Zambia (51.8%) [22], and Quatar (52.6%) [27] in lymphoma patients. Higher EBV viral loads were observed in studies conducted in Pakistan (75.3%) [28] and Libya (77.7%) [29] on different types of lymphoma. Studies conducted in African countries like Egypt [30] and Eretria [31] on FFPE samples exhibited a lower EBV prevalence. In these study sites, the EBV was detected in 38% and 27.8% of patients, respectively. Another study in China [32] also revealed a 24% EBV prevalence. These differences could be attributed to geographical variation and differences in the study participant groups and sample types (FFPE, blood and tissue biopsy).

Furthermore, our study revealed that 59.3% of HL patients and 54.8% of NHL patients exhibited a higher EBV viral load. Similar findings were observed in Zambia [22]. However, variations in EBV viral load among HL and NHL patients were observed in other studies conducted in different regions such as France [33], Iran [34,35], Libya [29], and Thailand [36]. These discrepancies across geographic locations may be attributed to differences in EBV genotypes [37], as well as environmental and dietary factors [38].

Additionally, our findings indicated that 67.6% of DLBCL patients had a higher EBV viral load compared to the other NHL groups. This prevalence is consistent with a study conducted in Libya (66.7%) [29], but higher than the rates reported in studies from France (5%) [33], Turkey (5.3%) [39], and Korea (14.3%). The variations in these findings could be attributed to differences in the methodologies used for detection (e.g., immunohistochemistry vs. RT qPCR) as well as variations in the sample types utilized in the studies.

Our study identified associations between EBV viral load and HIV status as well as among the different study participants (sample type). Similar associations between EBV viral loads and these variables have been observed in studies conducted in Switzerland [40], Argentina [41], and the USA [42]. However, no significant associations were found between EBV viral loads and other sociodemographic characteristics (age, sex, and lymphoma type) in our study. Comparable findings regarding the association of the EBV with sociodemographic factors were reported in studies conducted in Qatar [27], Iran [43], and Zambia [22]. However, studies conducted in England revealed an association between EBV status and age group [44]. Additionally, another study in the United Kingdom demonstrated variations in EBV prevalence with age and sex [45]. These differences could be attributed to variations in study population, sample types, and geographic locations.

## 5. Limitations of the Study 

The limitations of this study include loss of follow-up and a lack of pathology results for some study participants with suspected lymphoma, leading to incomplete data analyses for a subset of cases and limited serological assay results, as only the EBV VCA IgG test was conducted, potentially meaning additional serological markers or antibody responses related to EBV infection were missed. Future studies could address these limitations by ensuring better follow-up and incorporating a broader range of serological assays for a more comprehensive understanding of EBV infection in lymphoma patients. Future directions for the study include conducting in vitro and in vivo experiments to explore the mechanistic aspects of EBV infection in lymphoma development. Additionally, performing whole-genome sequencing or targeted next-generation sequencing can help investigate genetic variations and viral strains of the EBV in lymphoma patients from Ethiopia.

## 6. Conclusions

In this study, we investigated the serological and molecular detection of the EBV in lymphoma patients in Ethiopia, making it the first of its kind in the country. The findings from this study provide important foundational information that can guide future research efforts within Ethiopia and be potentially extended to other regions in East Africa. The findings suggest the importance of considering EBV viral loads in lymphoma patients, particularly in relation to HIV status. Further research is needed to explore the implications of these findings on disease progression, treatment response, and clinical outcomes in the Ethiopian population. Overall, this study contributes to our understanding of EBV infection and its association with lymphoma in Ethiopia, providing valuable insights for future research and clinical management of patients. Building upon these findings and conducting broader research will enhance our knowledge on the EBV’s impact and aid in the development of effective prevention and control measures.

## Figures and Tables

**Table 1:** Demographic and clinical characteristics of the study participants.

Characteristics	Number	Percent
Study participants (*n* = 305)		
FFPE blocks	133	43.6
Confirmed lymphoma subjects	120	39.3
Suspected lymphoma subjects	52	17
Sex		
Male	197	64.6
Female	108	35.4
Age at diagnosis		
<20	50	16.4
21–30	58	19.0
31–40	59	19.3
41–50	69	22.6
51–60	34	11.1
>61	32	10.5
Not applicable	3	1
HIV status (*n* = 172)		
Positive	29	16.9
Negative	122	70.9
Unknown	21	12.2
Lymphoma types (*n* = 305)		
Hodgkin’s lymphoma	91	29.8
Non-Hodgkin’s lymphoma	197	64.6
Non-lymphoma (from suspected subjects)	17	5.6

**Table 2:** Associations of sociodemographic and clinical data with EBV serology.

Characteristics (*n* = 150)	VCA IgG Negative (*n*)	VCA IgG Intermediate (*n*)	VCA IgG Positive (*n*)	Total (*n*)	*p*-Value
Age					0.56
<20 years	1	0	15	16	
21–30 years	0	0	37	37	
31–40 years	0	1	32	33	
41–50 years	2	0	36	38	
51–60 years	1	0	12	13	
>60 years	1	0	12	13	
Sex					0.31
Male	4	0	92	96	
Female	1	1	52	54	
HIV status					0.63
HIV positive	0	0	26	26	
HIV negative	5	1	100	106	
Unknown	0	0	0	18	
Lymphoma type					0.64
Hodgkin’s lymphoma	1	0	48	49	
Non-Hodgkin’s lymphoma	4	1	96	101	

**Table 3:** The distribution of EBV viral load among lymphoma patients in Ethiopia.

Characteristics	<5000 EBV Copies/mL	5000–10,000 EBV Copies/mL	≥10,000 EBV Copies/mL	Total	*p*-Value
Study participants (*n* = 288)					<0.01
FFPE blocks	77 (57.8%)	14 (10.5%)	42 (31.6%)	133	
Lymphoma patients	3 (2.5%)	30 (25%)	87 (72.5%)	120	
Suspected lymphoma patients	0 (0%)	2 (5.7%	33 (94.3%)	35	
Age					0.78
<20 years	18 (37.5%)	9 (18.7%)	21 (43.8%)	48	
21–30 years	8 (15.1%)	6 (11.3%)	39 (73.6%)	53	
31–40 years	15 (28.3%)	11 (20.7%)	27 (51%)	53	
41–50 years	16 (23.9%)	7 (10.4%)	44 (65.7%)	67	
51–60 years	12 (36.4%)	7 (21.2%)	14 (42.4%)	33	
>60 years	10 (32.2%)	6 (19.3%)	15 (48.4%)	31	
Not applicable	1 (33.3%)	0 (0%)	2 (66.7%)	3	
Sex					0.97
Male	53 (27.7%)	31 (16.2%)	107 (56%)	191	
Female	27 (27.8%)	15 (15.5%)	55 (56.7%	97	
HIV status					<0.01
HIV positive	0 (0%)	4 (14.3%)	24 (85.7%)	28	
HIV negative	3 (2.7%)	26 (23.9%)	80 (73.4%)	109	
Unknown	77 (51%)	16 (10.6%)	58 (38.4%)	151	
Lymphoma type					0.65
Hodgkin’s lymphoma	22 (24.2%)	15 (16.5%)	54 (59.3%)	91	
Non-Hodgkin’s lymphoma	58 (29.4%)	31 (15.7%)	108 (54.8%)	197	

## Data Availability

Available upon request.
